# Response of Human Immunodeficiency Virus-Associated Cerebral Angiitis to the Combined Antiretroviral Therapy

**DOI:** 10.3389/fneur.2017.00095

**Published:** 2017-03-13

**Authors:** Julian Cheron, Chloé Wyndham-Thomas, Niloufar Sadeghi, Gilles Naeije

**Affiliations:** ^1^Department of Neurology, Erasme University Hospital, Université Libre de Bruxelles, Brussels, Belgium; ^2^Immunodeficiency Treatment Unit, Erasme University Hospital, Université Libre de Bruxelles, Brussels, Belgium; ^3^Department of Neuro-Radiology, Erasme University Hospital, Université Libre de Bruxelles, Brussels, Belgium

**Keywords:** human immunodeficiency virus, brain vasculitis, cerebral angiitis, HIV-associated angiitis, vessel wall imaging

## Abstract

When secondary causes are excluded, mechanisms underlying central nervous system angiitis (ACNS) in human immunodeficiency virus (HIV)-infected patients are still not understood and optimal treatment remains undefined. We report here a patient with an untreated HIV infection who presented multiple ischemic strokes probably due to HIV-ACNS. ACNS signs on vessel-wall imaging magnetic resonance monitoring retracted with combined antiretroviral therapy without adjunct immunosuppressive drugs.

## Introduction

Between 1 and 5% of patients carrying the human immunodeficiency virus (HIV) will present a stroke and up to 30% will display a cerebral ischemic lesion on autopsy. In these cases, stroke can be a consequence of opportunistic infections, coagulation abnormalities, meningitis, and vasculitis. However, whether HIV-infected patients are predisposed to increased risk of stroke by a direct or indirect virus-associated vascular change or because of the atherogenic effect of antiretroviral agents remains unclear (1, 2).

Angiitis of the central nervous system (ACNS) is one of the main atiologies of ischemic strokes in HIV-positive patients been accounted for 13–28% of the cases. ACNS in a HIV-positive patient can be secondary (to opportunistic infections) or due to HIV itself either directly or indirectly through modifications in the local immune and inflammatory response. Frequently, the vasculopathy is present without any other discernible disease leading to the diagnosis of HIV-associated ACNS (HIV-ACNS) (3, 4). HIV-ACNS cases were mainly reported before the era of combined antiretroviral therapy (c-ART) and almost every pattern of vasculitis (inflammatory cells in the blood vessel wall together with associated wall damage) of small, medium, and large vessels have since been reported (5). Some authors deem HIV-ACNS to be immune mediated and akin to primary angiitis of the CNS (3, 6) while others consider HIV-ACNS to be caused by a direct action of the HIV (5, 7, 8). A main argument for the direct involvement of HIV in HIV-ACNS is the identification of HIV particles (by RNA *in situ* hybridization and electron microscopy) in the perivascular tissues of patients with HIV-ACNS (5). However, this evidence is counterbalanced by the histological identification of immune deposits in the vessel walls of the same patients (5).

The optimal treatment for HIV-ACNS remains unknown as only a limited number of case reports are available (9–13). In the majority of cases, the introduction of an immunosuppressive therapy [extrapolated from evidence from PACNS’s studies (14)] is associated with HIV antiretroviral therapy to cover both alleged physiopathological mechanisms of HIV-ACNS. However, restoring immunity with c-ART on one hand and inducing immunosuppression on the other hand with immunosuppressive drugs seems paradoxical and precludes clear understanding of the underlining pathophysiology when patients improve.

Here, we present the case of a 56-year-old man with an untreated HIV infection presenting multiple ischemic strokes due to a probable case of HIV-ACNS. Vasculitis signs on serial vessel-wall imaging (VWI-MR) retracted with c-ART without adjunct immunosuppressive drugs suggesting that the pathophysiology of HIV-ACNS is more likely being directly related to the HIV itself than to a immune reaction.

## Background

A 56-year-old man admitted at the emergency room (ER) with a recurrent left leg weakness occurring for more than 24 h. The individual chart indicated the presence of HIV-1 infection since 2012 but without antiretroviral treatment. Anamnesis revealed that the HIV was sexually transmitted and that the patient had never consumed drugs of abuse. On admission, the CD4+ cell count was 313/mm^3^ (15.69% lymphocytes) and HIV-1 viral load was 59,375 copies/ml.

Additionally, the patient had a history of syphilitic aortic aneurysm treated by Bentall surgery 10 years ago for which he is treated with an anti-vitamin K (acenocoumarol, dose adjusted as necessary) and concomitantly presents multiple cardiovascular risk factor including obesity (BMI = 35), glucose intolerance, arterial hypertension, and hypercholesterolemia treated by amlodipine and simvastatin.

The weakness in his left leg was first noticed while driving. He went to the ER where he recovered completely within 6 h and was discharged after an unremarkable brain computed tomography angiography. The next day, the patient felt again a left leg weakness and was admitted a second time at the ER.

**Figure 1:**
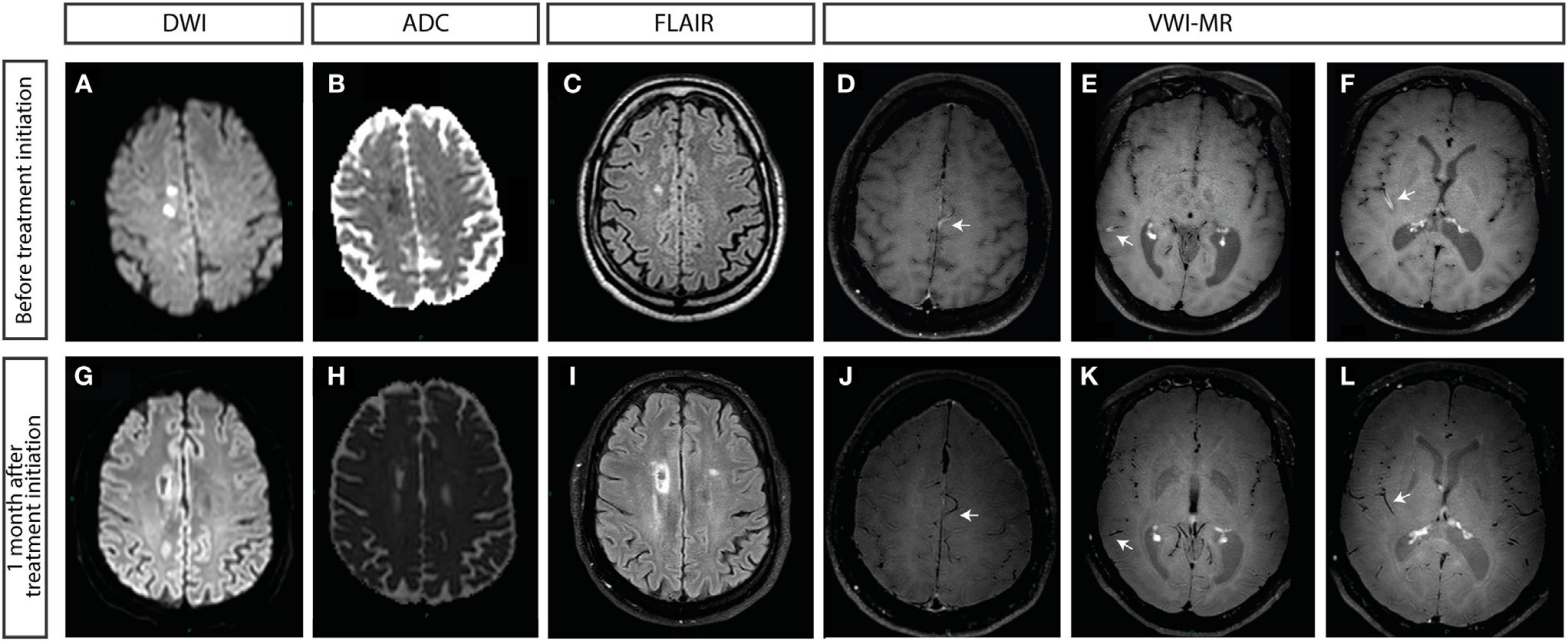
**Brain MRI at admission (A–F) and 1 month after treatment initiation (G–L)**. **(A,G)** Diffusion-weighted imaging and **(B,H)** apparent diffusion coefficient showing restricted diffusion in the right anterior cerebral artery territory, compatible with a recent ischemic stroke. **(C)** FLAIR sequence showing postischemic lesions in the anterior territory. **(I)** Extension of the lesion in FLAIR confirming established lesions in right and left anterior cerebral arteries. **(D–F,J–L)** Vessel wall imaging with MRI (VWI-MR), black blood MRI [high-resolution (3-T), black-blood fat suppressed pre- and post-contrast T1 weighted images]. **(D–F)** Concentric contrast enhancements (CEs) suggesting a central nervous system vasculitis (indicated by arrows). **(D)** CE of a distal branch of the left anterior cerebral artery. **(E,F)** CE of distal branches of the right middle cerebral artery. **(J–L)** Retraction of the CE after 1 month of combined antiretroviral treatment.

On this second admission, the general physical examination was within normal limits. Neurologic examination disclosed a left leg-predominant hemiparesis sparing the face associated with pyramidal syndrome. Brain MRI indicated restricted diffusion in the right anterior cerebral artery territory, compatible with a recent ischemic lesion, and lacunar lesions in the left temporo-occipital and right superior frontal region (Figure 1). Such atypical stroke pattern led us to perform a VWI-MR that demonstrated multiple vascular wall contrast enhancement (CE), particularly on distal branches of the middle (Figures 1E,F) and anterior cerebral artery (Figure 1D). Cerebrospinal fluid (CSF) analysis disclosed normal cell count and biochemistry. In addition, extensive blood tests and serial CSF studies were normal. Syphilitic tests showed a negative VDRL and positive TPHA in the blood while these two tests were negatives in the CSF. In the CSF, bacterial, mycobacterial, fungal, and viral cultures were negatives.

The combination of HIV infection and multiple ischemic strokes in different arterial territories associated with the enhancement of cerebral wall vessels both at the stroke territory and at other arteries emerging from Willis polygon led to the diagnosis of HIV-ACNS. cART (dolutegravir/abacavir/lamivudine) was immediately introduced but without adjunct immunosuppressive therapy. This was decided based on the patient’s immune status, the immediate and complete neurological recovery of this initial episode, and the absence of recurrence in the 4 days between the patient’s admission and the VWI-MR results.

After the first month, most vessels did not show CE anymore (Figures 1J–L). After 8 months, there was no more CE visible on VWI-MR. This clinical course is highly suggestive of isolated CNS vasculitis secondary to HIV infection. Unfortunately, 11 months after the initial event and 3 months after a VWI-MR devoid of vasculitic activity, the patient presented a right sylvian stroke. In fact, the INR was measured below the therapeutic threshold required for patients who underwent aortic root replacement such as the Bentall procedure [INR: 1.72 where it should be kept between 2.5 and 3 (15)] and an extensive cardiovascular workup (transthoracic and transesophageal echocardiography, carotid ultrasonography, 72-h electrocardiogram), which did not disclose any intracardiac thrombus, valvular vegetation, carotid macroangiopathy neither cardiac arrhythmia. In addition, reactivation of vasculitic activity was not detectable on a new VWI-MR acquisition.

This study was carried out in accordance with the recommendations of Erasme University Hospital ethics committee with written informed consent from the patient for the publication of this case report.

## Discussion

We reported here a case of a probable HIV-ACNS whose evolution was favorable while treated only with c-ART. The case also illustrates the potential contribution of VWI-MR to the diagnosis of ACNS and the monitoring of disease during treatment.

The patient was admitted at the hospital with a recurrent left leg weakness occurring over 24 h, without neither headache nor alteration in vigilance, which are both usually seen as classical clinical manifestations of PACNS but only found in 54 and 27%, respectively, of patients (16). When headache is present, its characteristics are variable and many authors consider that thunderclaps headaches as not characteristic of CNS vasculitis but that could suggest, for example, a reversible vasoconstriction syndrome (RCVS) (17). Nevertheless, a focal neurologic deficit is found in 83% of PACNS patients and could be the only symptom of HIV-ACNS (14). MR brain imaging of this HIV-positive patient with sudden-onset focal signs was essential both for the early diagnosis of ischemic stroke and for the diagnostic workup. Once the diagnosis of stroke was confirmed, the clinical workup focused on both conventional causes of stroke and also on HIV-associated causes. Briefly, the patient had been treated with a well-dosed anti-vitamin K drug, protecting him from cardioembolic events. Opportunistic infections leading to stroke were unlikely, considering relatively high CD4+ level, normal serial extensive blood tests, and normal serial cerebrospinal fluid studies. For example, VZV infection and tertiary syphilis were implausible in front of a normal CSF cell count, biochemistry, and index. Although the patient had many cardiovascular risk factors, an atheroembolic origin was ruled out since the results from doppler sonography of the supra-aortic vessels and magnetic resonance angiography were normal. Likewise, a microangiopathic origin could have been suspected but was dismissed by the MRI lesions localization.

The gold standard for the diagnosis of CNS vasculitis is histopathological evidence of vessel wall inflammation. However, a brain biopsy is an invasive technique and may reveal false negative findings, particularly in patients with predominant or isolated proximal large artery involvement (7). Thus, frequently, the diagnosis is based on a clinico-radiologic pattern suggestive of the disease. MRI has gained value in this field with the development of specific sequences dedicated to vessel walls and its inflammation through vessel walls CE detection (18–20).

The CE is visualized with a particular technique based on a 3DT1-weighted TSE sequence (Black Blood), which better differentiate the vessel wall from the lumen blood signal.

Recently, many studies tried to evaluate the performances of this technique in PACNS diagnosis and demonstrated that VWI could differentiate vasculitis from other vasculopathy-like atherosclerosis and RCVS offering an excellent specificity (18, 19). The VWI-MR of the patient was not suggestive of RCVS as studies (20, 21) highlight the lack of CE in all included RCVS patients. Nonetheless, the patient had many cardiovascular risk factors (including HIV infection) and an eventual resulting intracranial atherosclerosis could have led to focal vessel wall CE. However, the patient’s CE characteristics were not suggestive of an atherothrombotic mechanism. Indeed, CE was distal, concentric, and visible on important lengths of the involved arteries but also on intracranial arteries (middle and posterior cerebral arteries) that did not irrigate the infarcted tissues, corroborating a vasculitic process (19). In a HIV-infected patient, relevant differential diagnoses should include HBV and HCV associated vasculitis. Indeed, those two viruses are linked to medium vessel vasculitis [such as hepatitis B-associated polyarteritis nodosa (22) and hepatitis C virus-associated cryoglobulinemia vasculitis with brain involvement (23)]. However, those diagnoses were reasonably excluded after HBV, HCV, and cryoglobulins tested negative.

An analogy could be drawn between the radiological patterns of PACNS and HIV-ACNS, particularly regarding vessel abnormalities. Nevertheless, this patient presented certain differences with the classical MRI abnormalities found in PACNS. Indeed, the patient lacked both the gadolinium meningeal enhancement on MRI that is observed in 40.3% of PACNS (14) and the intracranial haemorrhagic lesions that are also commonly found (16). CSF studies have showed both low sensitivity and specificity for the discrimination of PACNS from secondary ACNS. However, normal CSF studies and negative testing for pathogens such as VZV, CMV, EBV, HBV, HCV, toxoplasmosis, *Salmonella* associated with ACNS in HIV patients argued for primary involvement of the HIV in the vasculopathy of the patient (7, 8).

Some authors have proposed, in case of vasculitis associated with HIV infection, to start a systemic immunosuppressive treatment in conjunction with c-ART. These recommendations are based on rare case reports (9, 11, 12) and were established by analogy to what has been done in large observational studies of PACNS (14, 16). However, Bhagavati and Choi in 2008 and Cutfield et al. in 2009 described two severely immunocompromised HIV-positive patients (CD4+ cell count = 3 and 94 cells/μl, respectively) where outcomes improved with cART alone (10, 24). As described above, the clinical situation of the patient and the result of ancillary investigations led us to choose this option. Close follow-up was planned with serial neurologic examination and VWI-MR. Here, c-ART alone led to a complete retraction of vessel wall CE. All these factors support the diagnosis of HIV-ACNS driven by HIV infection.

## Concluding Remarks

To the best of our knowledge, this is the first report demonstrating a complete retraction of vessel wall CE after cART initiation without additional immunosuppressive treatment in a so far cART-naive HIV patient. It highlights the potential role of dedicated wall vessel sequences on brain MRI for the diagnosis and follow-up of HIV-ACNS and its mimics. Further studies are required to confirm these preliminary results. The poor outcome, despite HIV-ACNS resolution, highlights the further complexity of the multiple comorbidities of HIV-infected patients.

## Author Contributions

JC: conception, collection of the data, drafting of the article, critical revision, and final approval. CW-T: internistic follow-up of the patient, revision, and final approval. NS: radiological follow-up of the patient, revision of the manuscript and figure, and final approval. GN: neurological follow-up of the patient, critical revision of the manuscript, and final approval.

## Conflict of Interest Statement

The authors declare that the research was conducted in the absence of any commercial or financial relationships that could be construed as a potential conflict of interest.
